# Complete chloroplast genome sequence of an ornamental grass *Muhlenbergia capillaries* (Poaceae: Chloridoideae)

**DOI:** 10.1080/23802359.2020.1764403

**Published:** 2020-05-13

**Authors:** Qi Wang

**Affiliations:** Yunnan Forestry Technological College, Kunming, Yunnan, China

**Keywords:** Chloridoideae, chloroplast genome, relationship

## Abstract

*Muhlenbergia capillaris* (Lam.) Trin. is an ornamental grass belonging to the genus *Muhlenbergia* Schreb. in the family Poaceae. To better understand its phylogenetic relationship with respect to the other species in the tribe Chloridoideae, the first complete chloroplast genome of *Muhlenbergia* was determined. The complete chloroplast genome of *Muhlenbergia capillaris* is 134,907 bp in length, consisting of one large single-copy (LSC) region of 80,175 bp, one small single-copy (SSC) region of 12,706 bp, and a pair of inverted repeat (IR) regions of 21,013 bp. The overall GC content of the genome is 38.1%. Further, maximum likelihood phylogenetic analysis with TVM + F+R3 model was conducted using 28 complete plastomes of the Poaceae, which support close relationships among species of *Muhlenbergia*, *Hilaria* Kunth, *Distichlis* Raf., and *Bouteloua* Lag., followed by those of *Tragus* Haller.

*Muhlenbergia capillaris* (Lam.) Trin., an ornamental grass distributed in Prairies, was assigned to the genus *Muhlenbergia* Schreb. in the family Poaceae. There are over 170 species in *Muhlenbergia* (http://www.plantsoftheworldonline.org/), and most of them are morphologically highly variable (Peterson et al. 1995). At the species level, the reported nuclear ITS and several chloroplast genomic markers were limited to resolve the phylogenetic and species identification problems in *Muhlenbergia* (Peterson et al. 2010). Here, we report the first complete chloroplast genome sequence of *Muhlenbergia.*

About 2 g leaf of *M. capillaris* in the nursery of Yunnan Forestry Technological College (Yunnan, China; Long. 102.771847°E, Lat. 25.097343°N, 1984 m) were collected for DNA extraction (Doyle and Dickson 1987). The voucher was deposited at the Yunnan Forestry Technological College (Accession Number: *HFTC-Qi Wang 002*). The complete chloroplast genome was sequenced following Zhang et al. (2016), and their 15 universal primer pairs were used to perform long-range PCR for next-generation sequencing. Clean paired-end reads were assembled with the GetOrganelle Kit (version 1.4.0) (Jin et al. 2018). The contigs were aligned using the publicly available chloroplast genome of *Hilaria cenchroides* Kunth (GenBank accession number KT168387) (Duvall et al. 2016) and annotated in Geneious 4.8.

The complete chloroplast genome of *M. capillaris* (SY001904), with a length of 134,907bp, was 73 bp and 1042 bp larger than that of *H. cenchroides*(134,824 bp, KT168387) and *Bouteloua curtipendula* (Michx.) Torr. (133,865 bp, KT168386) (Duvall et al. 2016). It was also 28 bp and 542 bp smaller than that of *Tragus australianus* S. T. Blake (134,935 bp, MK590077) and *Distichlis bajaensis* H. L. Bell (135,452 bp, KT168394) (Duvall et al. 2016; Anderson et al. 2019). The length of the large single-copy (LSC), inverted repeats (IRs), and small single-copy (SSC) regions of *M. capillaris* was 80,175 bp, 21,013 bp, and 12,706 bp, respectively. The overall G + C content was 38.1%.

Based on 28chloroplast genome sequences, we reconstructed a phylogenetic tree (Figure 1) to confirm the relationship between *M. capillaris* and other related species with published chloroplast genomes in Chloridoideae, with *Centropodia glauca* (Nees) Cope as an outgroup. Maximum likelihood (ML) phylogenetic analyses were performed based on TVM + F+R3 model in the iqtree version 1.6.7 program with 1000 bootstrap replicates (Nguyen et al. 2015). The ML phylogenetic tree with 59–100% bootstrap values at each node supported that close relationships among the species of *Muhlenbergia*, *Hilaria* Kunth, *Distichlis* Raf., and *Bouteloua* Lag., followed by *Tragus australianus*.

**Figure 1.**
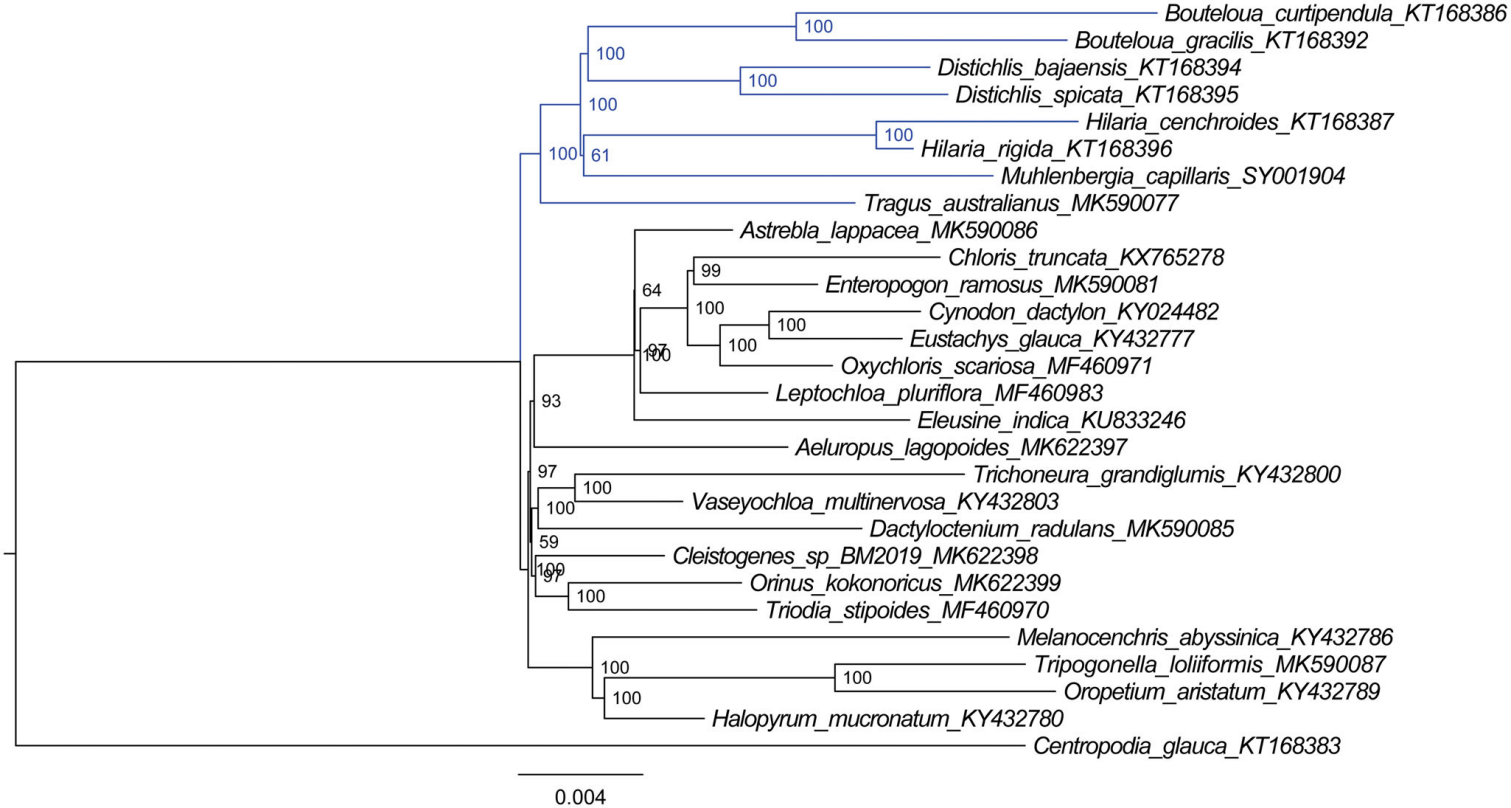
The ML phylogenetic tree for *M. capillaris* based on other 27chloroplast genomes of species in the family Poaceae.

## Data Availability

The chloroplast genome data of the *Muhlenbergia capillaries* will be submitted to Chloroplast Genome Database (https://lcgdb.wordpress.com). Accession numbers are SY001904.
